# Epigenetic signatures of alcohol abuse and hepatitis infection during human hepatocarcinogenesis

**DOI:** 10.18632/oncotarget.2444

**Published:** 2014-09-08

**Authors:** Ryan A. Hlady, Rochelle L. Tiedemann, William Puszyk, Ivan Zendejas, Lewis R. Roberts, Jeong-Hyeon Choi, Chen Liu, Keith D. Robertson

**Affiliations:** ^1^ Department of Molecular Pharmacology and Experimental Therapeutics, Mayo Clinic, Rochester, MN, USA; ^2^ Cancer Center, Georgia Regents University, Augusta, GA, USA; ^3^ Department of Pathology, Immunology and Laboratory Medicine, University of Florida, Gainesville, FL, USA; ^4^ Department of Surgery, University of Florida, Gainesville, FL, USA; ^5^ Division of Gastroenterology and Hepatology, Mayo Clinic, Rochester, MN, USA

**Keywords:** hepatocellular carcinoma, cirrhosis, etiology, epigenetics, DNA methylation

## Abstract

Hepatocellular carcinoma (HCC) is the second most common cause of cancer deaths worldwide. Deregulated DNA methylation landscapes are ubiquitous in human cancers. Interpretation of epigenetic aberrations in HCC is confounded by multiple etiologic drivers and underlying cirrhosis. We globally profiled the DNA methylome of 34 normal and 122 liver disease tissues arising in settings of hepatitis B (HBV) or C (HCV) viral infection, alcoholism (EtOH), and other causes to examine how these environmental agents impact DNA methylation in a manner that contributes to liver disease. Our results demonstrate that each ‘exposure’ leaves unique and overlapping signatures on the methylome. CpGs aberrantly methylated in cirrhosis-HCV and conserved in HCC were enriched for cancer driver genes, suggesting a pathogenic role for HCV-induced methylation changes. Additionally, large genomic regions displaying stepwise hypermethylation or hypomethylation during disease progression were identified. HCC-HCV/EtOH methylomes overlap highly with cryptogenic HCC, suggesting shared epigenetically deregulated pathways for hepatocarcinogenesis. Finally, overlapping methylation abnormalities between primary and cultured tumors unveil conserved epigenetic signatures in HCC. Taken together, this study reveals profound epigenome deregulation in HCC beginning during cirrhosis and influenced by common environmental agents. These results lay the foundation for defining epigenetic drivers and clinically useful methylation markers for HCC.

## INTRODUCTION

Hepatocellular carcinoma (HCC) accounts for ~80% of liver cancers with over three quarters of a million new cases diagnosed each year, establishing HCC as the fifth most prevalent form of cancer in males with the second highest mortality rate world-wide [1, 2]. In the United States, the majority of HCCs are a repercussion of chronic infection with hepatitis B virus (HBV), hepatitis C virus (HCV), chronic alcohol abuse (EtOH), and non-alcoholic steatohepatitis (NASH; fatty liver). A variety of minor etiologies also contribute, including primary biliary cirrhosis and hemochromatosis [3, 4]. Furthermore, the incidence of HCC in the U.S. has more than doubled in the last three decades, primarily due to the increase in prevalence of chronic HCV infection and the rise in obesity and association of diabetes and red meat/fat consumption with HCC [5-8]. Liver cancer deaths are expected to continue to grow, surpassing breast, prostate, and colorectal cancer to become the third most prevalent cause of cancer mortality world-wide by the end of the 2020s [9]. Importantly, prolonged liver damage associated with these conditions is manifested by cyclical extirpation and regeneration of hepatocytes, leading to a multistep process of inflammation, progressing fibrosis, cirrhosis, and ultimately carcinogenesis, with upwards of 90% of all HCC occurring within cirrhotic liver [10, 11]. Due to the frequent co-occurrence of cirrhosis with hepatocellular carcinoma, treatment of patients with these liver diseases remains complex. Current treatments for early stage HCC patients include liver transplantation, surgical resection, and tumor chemoembolization [12]. Only 20-30% of patients, however, are diagnosed early enough for these treatments to be feasible, and in the presence of HCV, up to 80% of patients exhibit tumor recurrence within five years of resection [13]. The prognosis for advanced HCC is extremely poor. The only available systemic treatment is sorafenib, which achieves only modest clinical benefit in a small number of patients. These findings emphasize the need to delineate disease progression based upon etiologic background to ultimately improve prognostic and therapeutic approaches.

Today, hepatitis B and C viral infections and chronic alcohol abuse account for the vast majority of HCCs worldwide. HBV infection induces the expression of a unique repertoire of proteins, including HBV protein X (HBx), which disrupts p53 activity and impacts a plethora of cell signaling pathways including JAK/STAT, NF-κB, and Wnt [14]. Integration of HBV DNA sequences into host DNA also results in the disruption of tumor suppressor gene expression and formation of oncogenic host-viral fusion proteins and RNAs that contribute to carcinogenesis [15, 16]. Importantly, the risk of HCC is augmented up to 15-fold in chronically infected HBV patients [17]. Unlike HBV, HCV is an RNA virus with primary virion production taking place on hepatocyte lipid membranes. HCV core protein immortalizes primary human hepatocytes and core protein expression in mice leads to HCC [18, 19]. Infection of hepatocytes with HCV also leads to many transcriptional changes (e.g. downregulation of p16, STAT3 upregulation) [20]. Similar to HBV, the risk of HCC in the presence of HCV infection is elevated, with 17-30% of cirrhotic patients progressing to HCC within five years [21]. Alcohol, increasing in global consumption yearly, is responsible for almost two million deaths per year [22]. Alcohol intake increases cancer risk of the mouth, pharynx, larynx, esophagus, liver, colon, rectum, and breast. Acetaldehyde, a key metabolite of ethanol, is a known carcinogen and heavy alcohol consumption increases HCC incidence by 3-10 fold. Acetaldehyde forms DNA adducts resulting in defects in DNA repair and alcohol metabolism also generates free radicals, which promote inflammation though oxidative stress. Ethanol exposure and HCV infection synergistically increase the risk of HCC [22]. Morphologically, liver disease developing in the setting of alcohol abuse or HCV/HBV infection are indistinguishable [23], yet it is likely these agents act in distinct ways, that may or may not converge on common pathways to promote liver carcinogenesis. The shared and unique impact on the epigenome due to exposure to these environmental agents and how they differentially promote liver disease remains largely unknown, but it is probable that different exposures uniquely influence the epigenome in the pathogenesis of chronic liver disease [24].

Atypical epigenetic landscapes, in the form of global DNA hypomethylation and promoter hypermethylation, are a hallmark of human cancer and culminate in genome instability and gene silencing, respectively. Alterations in the epigenetic machinery in HCC, such as up-regulation of DNA methyltransferases (DNMTs), have been well established [25-27]. Indeed, hypermethylation of tumor suppressor genes in HCC occurs at a variety of loci (e.g. RASSF1, p16^INK4a^, E-cadherin). Furthermore, expression of DNMT3B4, a catalytically inactive splice variant of DNMT3B, is overexpressed in HCC and correlates with hypomethylation of pericentromeric satellite regions that may subsequently lead to chromosome instability [28]. Premalignant lesions (i.e. cirrhosis) also present with DNA methylation defects (e.g. GSTP1 promoter hypermethylation) [29-31]. In addition, S-adenosylmethionine (SAM), the methyl donor for DNA methyltransferases, is reduced in liver disease and supplemental SAM is suggested to be protective against HCC, highlighting the importance of DNA methylation in liver disease [32]. While it is known that HBV, HCV, and alcohol impact the epigenome to varying extents, the link between environmental agents and DNA methylation in liver disease is still poorly understood. For example, HCV core protein upregulates DNMT1/DNMT3B and causes epigenetic silencing of SFRP1, which is associated with increased HCC aggressiveness. DNMT upregulation then epigenetically silences E-cadherin and p16^INK4A^ expression [20, 25, 33]. Similarly, HBx increases DNMT activity, leading to hypermethylation of tumor suppressor genes [34, 35]. Overall, aberrant DNA methylation resulting from HBV, HCV, or alcohol exposure correlates with specific epigenetic defects, suggesting that each etiology exhibits both shared and distinct epigenetic features and should be approached as individual diseases [29]. Candidate-gene differential methylation studies have unveiled potential disease drivers for liver cancer [31]. Unbiased global methylation screening, however, has the potential to reveal epigenetic changes with high sensitivity and specificity to disease stage that may also represent epigenetic drivers of tumorigenesis.

Advanced HCC is highly lethal with limited effective treatment options. Consequently, there is a major unmet need for sensitive and reliable assays to detect early-stage disease. Furthermore, mutations in key cancer driver genes account for only a fraction of HCC cases (~2 mutations/megabase, [36]), highlighting the potential importance of the epigenome. In this study, we investigated the impact of alcohol and viral infection on the hepatocyte DNA methylome using the Infinium HumanMethylation450k BeadChip in a panel of 156 primary liver samples and 25 cultured liver cell types. The goal of this study was to define the variation in DNA methylation throughout liver cancer progression under the influence of environmental factors to infer distinct subclasses of cirrhosis and HCC based on epigenetic signatures. The results demonstrate clearly defined methylation patterns unique to normal liver, cirrhosis, and HCC. These patterns are accompanied by unique etiologic-driven DNA methylation signatures dependent upon disease stage. Furthermore, changes to the DNA methylome in HCC are primarily confined to large domains exhibiting coordinated hypomethylation or hypermethylation events. Analysis of cryptogenic HCC showed substantial overlap with HCC-HCV and HCC-EtOH, while metastases to the liver and biliary tumors reveal tissue/disease-dependent changes in DNA methylation. To our knowledge, a comprehensive genome-wide study of multiple etiologies and stages of HCC has not been performed to date. Results from this study are expected to pave the way for using epigenetic signatures to stratify patients for different treatment regimens and better define epigenetic drivers of this disease.

## RESULTS

### Categorization of patient samples

**Figure 1:**
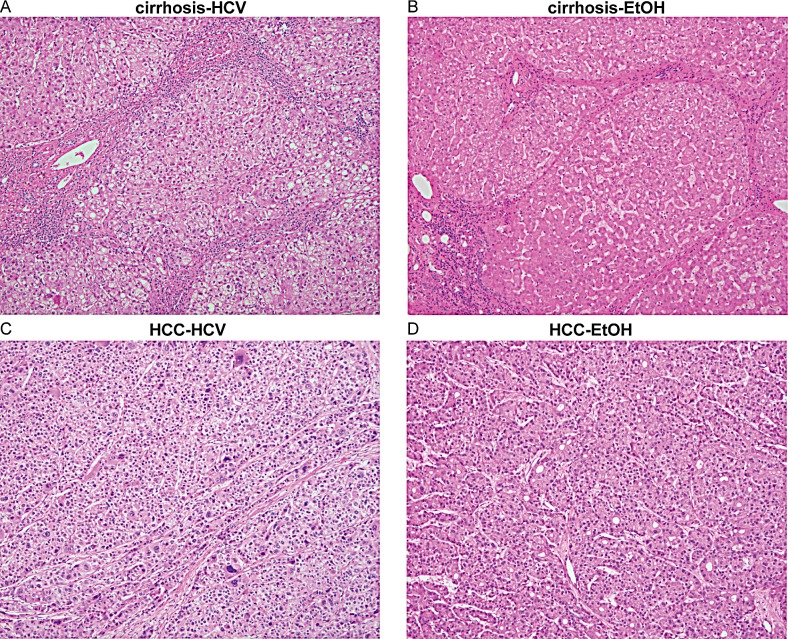
Histology of cirrhotic and hepatocellular carcinoma tissues Histological cross-sections of representative liver tissue stained with H&E from cirrhosis-HCV (A), cirrhosis-EtOH (B), HCC-HCV (C), and HCC-EtOH (D) at 100x magnification.

The total primary tissue pool for this study included 34 normal liver samples, 77 cirrhotic liver and 45 hepatocellular carcinomas, for a total of 156 primary human samples (Table 1). Cirrhosis samples were primarily categorized by HCV-infection (51%), chronic alcoholism (26%), and HBV-infection (8%), with the remaining group (Other) consisting of rare etiologies and cryptogenic cirrhosis (14%). HCC samples were similarly diverse, with 25% HCV, 4% HBV, 33% alcohol-related, and 38% resulting from other etiologies and cancers metastasizing to the liver (Table 1, Supplemental Table 1,2). Histopathologic analysis was performed and relevant clinical parameters are shown in Table 1. Representative examples of the histology of cirrhotic liver and HCC in the setting of HCV or alcohol are shown in Figure 1. These serve to emphasize that cirrhosis and HCC arising from different etiologies cannot be distinguished solely based upon histological features [23]. Global DNA methylation patterns were profiled for all samples utilizing the Illumina Infinium 450k HumanMethylation BeadChip (450k array). This allowed for assessment of the methylation status of up to 485,513 CpG dinucleotides across the genome (473,864 sites corresponding to allosomes, which are the focus of the current manuscript).

**Table 1:** Clinical features of human samples analyzed in this study

Clinical Feature	Criteria	HCV (n)	HCV (%)	EtOH (n)	EtOH (%)	Total (n)	Total (%)
Differentiation	1	4	33	6	40	10	37.04
	2	7	58	7	47	14	51.85
	3	1	8	2	13	3	11.11
Gender	Male	9	75	12	80	21	77.78
	Female	3	25	3	20	6	22.22
TNM Stage	T1N0Mx	4	33	6	40	10	37.04
	T2N0Mx	4	33	3	20	7	25.93
	T3N0Mx	4	33	5	33	9	33.33
	T4N0Mx	0	0	1	7	1	3.70
Tumor multifocality	yes	4	33	9	60	13	48.15
	no	8	67	6	40	14	51.85
Vascularization	yes	6	50	8	53	14	51.85
	no	6	50	7	47	13	48.15
Tumor size	>5 cm	4	33	7	47	11	40.74
	<5 cm	8	67	8	53	16	59.26
Cirrhosis	yes	12	100	14	93	26	96.30
	no	0	0	1	7	1	3.70

### Unveiling the impact of environmental exposures during liver cirrhosis

**Figure 2:**
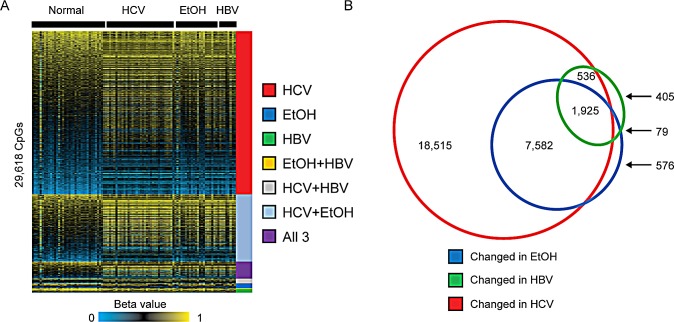
Early epimutations in cirrhosis are related to etiologic exposure A. Heatmap depicting beta values (β) for normal liver (n=34) or cirrhotic livers from HCV-infected individuals (HCV, n=39), chronic alcoholics (EtOH, n=21) or HBV-infected individuals (HBV, n=6). A color bar is shown with low methylation in blue, intermediate in black, and high methylation in yellow (FDR<0.05, Δβ>|0.1|). Samples are clustered based upon the groups into which they fall. CpGs were considered common if they were statistically significantly changed in more than one group. B. Venn diagram depicting the unique and overlapping CpG site changes in cirrhotic relative to normal liver using a change in β of at least 0.1.

We first interrogated the DNA methylation changes that occurred in liver cirrhosis, compared to normal liver, under conditions of chronic HBV or HCV infection or alcohol abuse. We identified 28,558, 10,162, and 2,945 aberrantly methylated CpGs in cirrhosis-HCV, cirrhosis-EtOH, and cirrhosis-HBV, respectively (Δβ>|0.1|, Figure 2A). Interestingly, the majority of CpG sites that showed an altered level of DNA methylation were unique to HCV infection (n=18,515 CpGs), while HBV infection (n=405 CpGs) and ethanol exposure (n=576 CpGs) showed relatively few distinct DNA methylation changes at this stringency level (Figure 2B). Principal component analysis of cirrhosis samples demonstrated clear separation between cirrhosis-HCV and normal liver, while HBV and EtOH exposure samples were less distinct relative to normal tissue (Supplemental Figure 1). Furthermore, the majority of methylation changes in alcoholic patients appeared to be shared with cirrhosis occurring in other etiologies (94%), suggesting that cirrhosis driven by ethanol abuse shares an overlapping epigenetic pathology with viral infection-induced cirrhosis.

### The effect of HCV and ethanol on the DNA methylome during liver carcinogenesis

**Figure 3:**
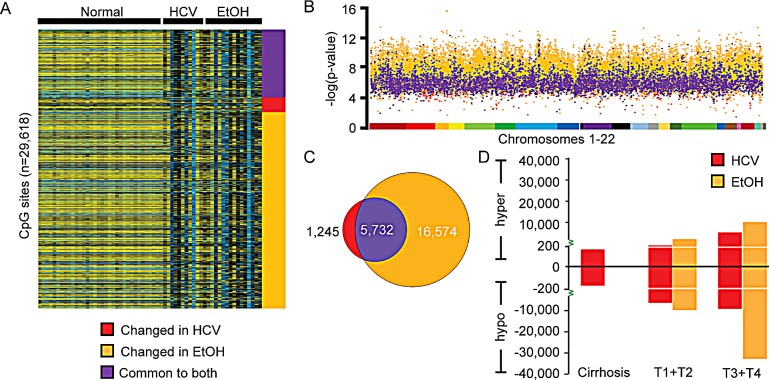
Ethanol exposure is the dominant epigenetic effector in late-stage liver disease A. Heatmap depicting the 18,257 CpGs whose methylation levels (β>|0.25|) are significantly different in HCC patient samples with hepatitis C infection (HCV) or in chronic alcoholics (EtOH) relative to normal liver. A color bar is shown to depict hypomethylation (blue) and hypermethylation (yellow) with intermediate methylation in black. B. Manhattan plot displaying changes specific to HCV infection (red), EtOH abuse (orange), or common to both (purple). Chromosomes 1-22 are color coded to demonstrate the distribution of methylation changes. C. Venn diagram depicting the unique DNA methylation changes found in HCC-HCV and HCC-EtOH, as well as conserved events between the two groups (Common to both). D. Bar chart depicting the number of DNA methylation changes in cirrhosis, TNM stage T1 and T2 (T1+T2) and TNM stage T3 and T4 (T3+T4) with a (Δβ>0.25, FDR<0.05) relative to normal liver.

The methylation status of 241,235 sites on the 450k array showed a significant change between control normal liver tissues and HCC. When a Δβ>|0.25| was applied to increase stringency, this narrowed the scope of the methylation change to 23,551 CpG dinucleotides (Figure 3A,B). As seen in Figure 3C, the majority of differentially methylated CpG sites were altered specifically in HCC-EtOH (n=16,574 CpGs), with roughly ten times fewer unique changes in HCC-HCV (n=1,245 CpGs) and a substantial portion (n=5,732 CpGs) overlapping between the two groups. Importantly, this trend was consistent at a variety of Δβ cutoffs and paired analysis of HCC and adjacent non-tumor tissue also supported heightened epigenetic deregulation in HCC-EtOH relative to HCC-HCV (data not shown). Furthermore, DNA methylation changes in both viral- and alcohol-induced HCC were dispersed throughout the genome, demonstrating a global alteration of methylation patterns (Figure 3B). Overall, HCC had drastically altered methylation profiles relative to normal and cirrhotic livers, with ethanol exposure impacting DNA methylation in HCC to a greater degree than HCV infection (Figure 3B,C). Importantly, the number of aberrant DNA methylation changes correlates with increased tumor stage (Figure 3D). Moreover, the methylation disparity between HCV and EtOH is exacerbated in later stages of hepatocarcinogenesis. This suggests that downstream of hepatocyte transformation, exposure to ethanol and/or its derivatives may be important for modulation of DNA methylation. These data therefore reveal massive epigenetic instability in HCC, suggesting that deregulation of DNA methylation plays a role in HCC progression and/or metastasis.

### Distribution of DNA methylation changes across genomic features

**Figure 4:**
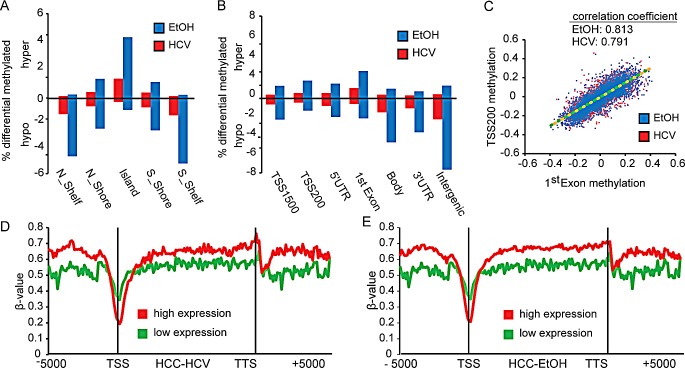
Distribution of DNA methylation changes across genomic features Bar charts depicting the relative percentage of DNA methylation changes at CpG islands (A) and intragenic (B) features (Δβ>0.25, FDR<0.05). Graphs above the x-axis depict hypermethylation, with hypomethylation events below. C. Dot plot showing the association between changes in TSS200 methylation and 1^st^ Exon regions in HCC-HCV (red) and HCC-EtOH (blue) relative to normal liver. Trend lines are shown for HCC-HCV (green dashed line) and HCC-EtOH (orange dashed line). Correlation coefficients are shown. DNA methylation β-values across genes including 5,000 base pairs flanking the transcription start site (TSS) and transcription termination site (TTS) of the gene for HCC-HCV (D) and HCC-EtOH (E) based upon highly expressed (red) and lowly expressed (green) genes defined from analysis of normal liver.

To examine the spatial distribution of methylation changes that occurred during liver disease progression, we annotated all CpG dinucleotides showing a significant change in DNA methylation based on their positional relationship to genes and CpG islands. The majority of hypermethylation changes in HCC in both the EtOH- and HCV-infection settings were positioned within CpG islands, while hypomethylation events occurred primarily outside of CpG islands (Figure 4A). Furthermore, increases in methylation were observed primarily flanking the transcription start site (TSS), while loss of methylation was distributed throughout the genome (intergenic) (Figure 4B, Supplemental Figure 2A, top graph). CpG sites within features near the TSS (i.e. TSS1500, TSS200, 5′UTR, 1^st^ Exon) were coordinately hypomethylated or hypermethylated in both HCC-HCV and HCC-EtOH, as is shown in TSS200 methylation and 1^st^ exon methylation plots (Figure 4C, Supplemental Figure 2B). Therefore, DNA methylation may be coordinately deregulated across large regions of DNA. The lack of inverse correlation between promoter and gene body methylation suggests that promoter hypermethylation and gene body hypomethylation act through alternate pathways to regulate gene transcription.

We next studied the relationship between methylation and gene expression using published microarray expression data derived from seven normal livers to determine the potential functional impact of DNA methylation on transcription [37]. In the top 25% most highly expressed genes in normal liver, there was markedly lower DNA methylation surrounding the TSS than in the 25% lowest expressed genes for normal liver, HCC-EtOH, and HCC-HCV (Figure 4D,E, Supplemental Figure 2A, bottom graph). Furthermore, the decrease in methylation flanking the TSS was accompanied by gains in methylation throughout the gene body (Figure 4D,E, Supplemental Figure 2A,B). This suggests that many genes with high promoter methylation and low gene body methylation are already lowly expressed in normal liver and vice versa. Interestingly, HCC-EtOH demonstrated clearer stratification of highly and lowly expressed genes based on DNA methylation patterns than HCC-HCV, implying greater disruption of the epigenetic machinery in this setting.

While cirrhotic samples showed fewer DNA methylation changes than HCC, the observed patterns in cirrhosis may reflect the DNA methylation status in HCC. Regions flanking CpG islands (CpG shores and shelves) were preferentially methylated during cirrhosis, suggesting that these regions prime CpG islands for hypermethylation and transcriptional silencing of their associated genes in HCC. Importantly, HCV-infection resulted in hypermethylation of CpG islands (CGIs) and genic features, while the milder DNA methylation changes in HBV infection and EtOH samples were constrained to hypermethylation of CGIs (Supplemental Figure 3A,B). Overall, the majority of DNA methylation changes in cirrhosis were hypermethylation events in response to HCV-infection, suggesting that the transition between cirrhosis-HCV and HCC may be a result of tumor suppressor gene hypermethylation following loss of epigenetic boundaries during cancer development. Therefore, DNA methylation changes during early liver disease may be the first step leading toward gene inactivation through hypermethylation in HCC.

### Functions of aberrantly methylated genes

**Figure 5:**
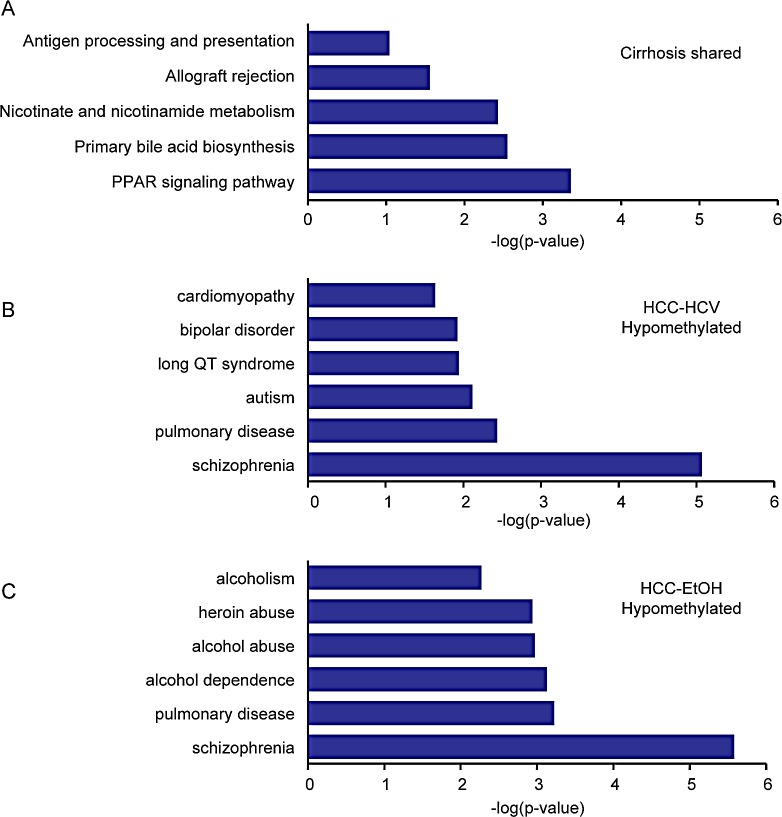
Ontology of differentially methylated genes in cirrhosis and HCC DAVID ontology for genes differentially methylated in HCV, HBV, and EtOH cirrhosis (A, Δβ>|0.1|), hypomethylated in HCC-HCV (B, Δβ<-0.25) or hypomethylated in HCC-EtOH (C, Δβ<-0.25). An expanded list of hypomethylated genes in HCC is shown in Supplemental Figure 4.

To better understand potential functional consequences of the DNA methylation changes that occur in cirrhosis and HCC, DAVID gene ontology analysis was performed for hyper- or hypomethylated CpGs in each etiology. Genes showing changes in DNA methylation that were conserved between two or more cirrhosis etiologies are primarily involved in pathways associated with apoptosis, antigen presentation, and immune responses, consistent with the inflammatory response that occurs during cirrhosis. Genes that are coordinately methylated in cirrhosis relative to normal liver, independent of etiology include CD74, which is highly expressed in the settings of inflammation and cancer (Figure 5A, Supplemental Table 4). Additionally, hypermethylated CpGs in HCC were associated with Wnt signaling, Hedgehog, and a variety of cancer subtypes, suggesting that DNA methylation plays a significant role in regulation of cancer-related pathways (Supplemental Table 5). Interestingly, hypomethylation events in chronic alcohol abuse patients were enriched in pathways of alcohol dependence and alcohol abuse, which were not present in HCC-HCV (Figure 5B,C, Supplemental Figure 4). This suggests that alcohol plays a role in a feedback loop that propagates the DNA hypomethylation phenotype, consistent with previous reports that MAT1A, which catalyzes the formation of SAM by linking methionine and ATP, is downregulated in liver disease leading to decreased SAM levels [38]. There appears to be direct involvement of epigenetic dysregulation within key cancer driving pathways in HCC, suggesting that aberrant DNA methylation is linked to the initiation and/or progression of liver disease.

### Identification of differentially methylated regions (DMRs)

**Figure 6:**
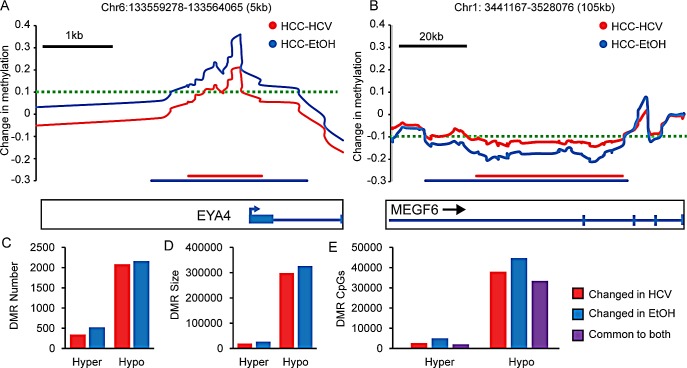
Differentially methylated regions (DMRs) in hepatocellular carcinoma Changes in DNA methylation in HCV infected and chronic alcohol abuse (EtOH) HCC samples relative to normal liver at the *EYA4* (A) and *MEGF6* (B) loci. The threshold for the DMR is shown by a dashed green line, and the DMR length is depicted by red (HCC-HCV) and blue (HCC-EtOH) lines. Schematic representations of the genes are shown below. The overall hypermethylated and hypomethyated DMR number (C), DMR size (D) and number of CpGs within DMRs (E) for HCV (red), EtOH (blue), and CpGs common to both groups of HCC samples (purple).

Differentially methylated regions (DMRs) are regions of the genome with large, coordinated methylation patterns that vary between different samples, tissues, or disease states [39]. These domains are thought to be important in the regulation of gene transcription, but have not been well studied, especially in HCC [40]. Global alterations of DNA methylation in HCC encompass 141,182 CpG sites (>|0.1|), roughly one quarter of all assayed CpGs. To determine if these CpGs were coordinately mis-regulated in DMRs, we grouped blocks of CpGs into DMRs if ten or more consecutive sites were consistently hypomethylated or hypermethylated (Δβ>|0.1|). Figure 6A and 6B illustrate two such regions (*EYA4* and *MEGF6* loci). *EYA4* is included within a hypermethylated DMR (~5,000bp), while *MEGF6* is in a hypomethylated DMR (~90,000bp). More than 30% of CpG dinucleotides significantly altered in HCC are conserved across large hypermethylated or hypomethylated domains. Importantly, the effects of alcohol on the methylome appear to be more substantial for both hypermethylated and hypomethylated DMRs. Consistent with the higher frequency of DNA methylation aberrations in HCC-EtOH relative to HCC-HCV, chronic alcoholic patients display more and larger DMRs (20,000 base pairs larger, on average), of both hyper- and hypomethylated subtypes (Figure 6C-E). Interestingly, we observed that a subset of these DMRs arise during cirrhosis and are enhanced in the magnitude of methylation change in HCC, exemplified by the HOXA and SUSD4 loci (Supplemental Figure 5A-C). A more focused analysis of one gene within the HOXA locus, *EVX1*, demonstrates that DNA hypermethylation encompassing a block of ~5,000bp is observed in cirrhosis (relative to normal), increases in HCC, and increases further still in HCC cell lines, (Supplemental Figure 5B). The stepwise DNA hypermethylation observed in the Infinium 450k data was confirmed using bisulfite genomic sequencing of the *EVX1* and *SUSD4* promoters in cirrhotic livers, cancerous liver samples, and the HCO2 cell line (Supplemental Figure 5B,C). This data demonstrates coordinated regulation of DNA methylation in HCC and suggests that maintenance of the epigenome at these loci is impaired in HCC. Indeed, these large blocks of hypermethylation could in part be due to erosion of epigenetic boundaries that are established during development. For example, loss of CTCF binding has been linked to hypermethylation of the *p16^INK4a^* promoter, while CTCF binding correlates with activation of *RASSF1A* and *CDH1*; *p16^INK4a^*, *RASSF1A* and *CDH1 are* targets of promoter hypermethylation and silencing in HCC [39, 41, 42].

### Conservation of epigenetic changes during liver disease progression

**Figure 7:**
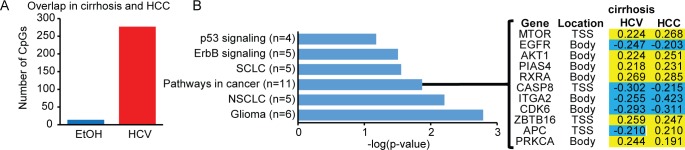
Conservation of cirrhosis-HCV DNA methylation changes in HCC A. Bar graph depicting the overlap of CpGs between cirrhosis-HCV (red) and cirrhosis-EtOH (blue) with HCC (p<0.05, β>|0.1|). B. Bar chart of DAVID gene ontology results for aberrantly methylated genes that overlap between cirrhosis-HCV and HCC. The resultant gene list, Δβ-values, and a heatmap is shown with hypermethylation events in yellow and hypomethylation events in blue (relative to normal). The CpG site location either near the transcription start site (TSS) or within the gene body (body) is listed.

Next, we compared DNA methylation changes in HCC to those occurring in cirrhotic liver samples (Figure 7A). While EtOH exposure samples showed relatively little overlap between cirrhosis and HCC, most likely due to the lower number of methylation changes in cirrhosis-EtOH, the HCV-infected samples showed a significant conservation of aberrantly modified CpGs in cirrhosis and HCC. DAVID ontology analysis revealed enrichment of cancer-associated pathways, including the well-characterized cancer genes *MTOR*, *EGFR*, *AKT1*, and *CASP8* in the region of overlap (Figure 7B). Interestingly, CpGs with altered DNA methylation were located near the transcription start site, as well as in the gene body. For example, the *CASP8* promoter was hypomethylated, while the gene body of *AKT1* was hypermethylated relative to normal tissue. While there is a firm link between promoter methylation and gene repression, the role of gene body methylation in regulating transcription, if any, is unknown. It has been noted, however, that methylation in the gene body is directly proportional to the level of transcription, suggesting that elevated gene body methylation may equate to increased gene expression, although this remains to be tested [43]. In addition, gene bodies may contain enhancers, whose epigenetic regulation could contribute to transcriptional regulation of the gene within which they are contained or other more distant genes. Gene bodies may contain non-coding RNAs that are capable of regulating transcription, such as the epigenetically regulated non-coding RNA *HOTAIR* in the HOXD locus [44]. Regardless of their location, these DNA methylation changes may represent useful markers of disease progression for identifying patients at elevated risk for HCC.

### Examination of DNA methylation changes in liver disease arising from less common etiologic exposures and genetic defects

**Figure 8:**
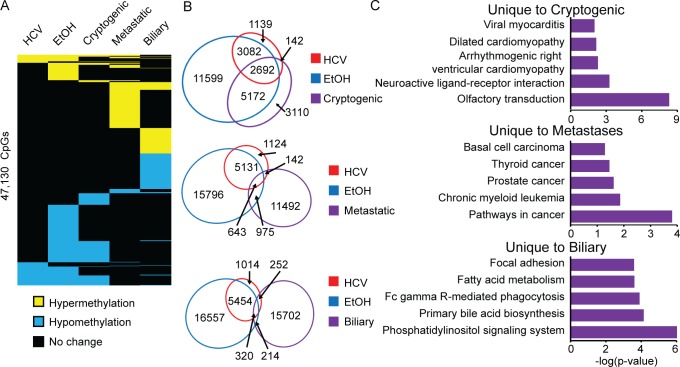
Diverse DNA methylation changes in liver-associated cancers A. Heatmap of statistically significant changes in hepatitis C infection (HCV), chronic alcoholic (EtOH), and cryptogenic HCC as well as metastases to the liver (Metastatic), and biliary tumors (Biliary) relative to normal liver. Hypermethylation is shown in yellow, hypomethylation in blue, and no change in black (p<0.05, Δβ>|0.25|). B. Venn diagrams of overlapping and non-overlapping DNA methylation changes with color-coded circles in HCC-HCV (red), HCC-EtOH (blue) and cryptogenic (top, purple), metastatic (middle, purple), and biliary (bottom, purple) tumors. C. DAVID gene ontology analysis for aberrantly methylated genes unique to cryptogenic (top), metastatic (middle), and biliary (bottom) tumors.

The majority of liver disease samples analyzed here are associated with chronic hepatitis viral infection and alcohol abuse, reflective of the incidence of these exposures in the United States. We have, however, banked a smaller number of liver disease samples arising from other exposures, genetic causes, or unknown etiology (cryptogenic). To obtain an initial assessment of how the DNA methylome is impaired in these other etiologies, and to gain additional evidence for shared and distinct DNA methylation changes between the rare and common risk factors (hepatitis infection, alcohol abuse), we performed Infinium 450k analysis on 37 such samples (n=11 cirrhosis, n=16 HCC, Supplemental Table 1,2). Furthermore, to assess the role of the liver microenvironment on DNA methylation patterns in cancer cells originating in different tissues, we profiled liver metastases arising from colon cancer, lymphoma, melanoma, sarcoma, squamous carcinoma, and neuroendocrine tumors for comparison with primary liver cancers. Few consistent changes were identified among all of these samples in cirrhosis or HCC, likely due to the heterogeneity of sample subtypes (data not shown). Therefore, we performed a more focused analysis of DNA methylation changes in specific liver disease sub-groups. Cryptogenic liver disease patients lack clear genetic, environmental, and epigenetic drivers [45]. To better understand these rare and poorly characterized samples, we profiled n=3 and n=6 for cirrhosis and HCC, respectively. We observed that in cryptogenic cirrhosis, relatively few changes in DNA methylation occured, while 11,116 CpGs displayed aberrant DNA methylation in cryptogenic HCC (Figure 8A, Supplemental Figure 6). Interestingly, 72% of these changes were conserved with HCC-HCV and/or HCC-EtOH, suggesting a shared epigenetically driven pathway for HCC (Figure 8B). In addition, metastases to the liver had relatively little overlap with HCC-HCV and HCC-EtOH, but a very large unique epigenetic profile (Figure 8B). Ontological analysis of CpGs aberrantly methylated in metastases showed enrichment for cancer pathways such as colorectal and thyroid cancer, which may reflect the tissue or disease of origin rather than the environment of the liver (Figure 8C, middle panel). Similarly, biliary tumors demonstrated more than 15,000 unique DNA methylation changes, whose associated genes are involved in pathways including bile acid biosynthesis (Figure 8B, C, lower panel). At present, few advanced HCC samples are available for further study as these patients are ineligible for surgical resection and liver biopsies are rarely performed at this stage; even fewer samples are available for low frequency etiologies (e.g. cryptogenic HCC). Nevertheless, our data demonstrate distinct epigenomic changes in each etiologic exposure linked to increased HCC risk that may be present at specific stages of tumor development, including cirrhosis. This raises an interesting question as to whether cancer stage-specific epigenomic signatures can be generated for HCC. Future studies will pursue this avenue by analyzing DNA methylation patterns using a larger number of HCC specimens that represent various tumor etiologies, grades, and clinical stages.

### DNA methylation changes in human primary tissue compared to cultured primary and HCC cell lines

**Figure 9:**
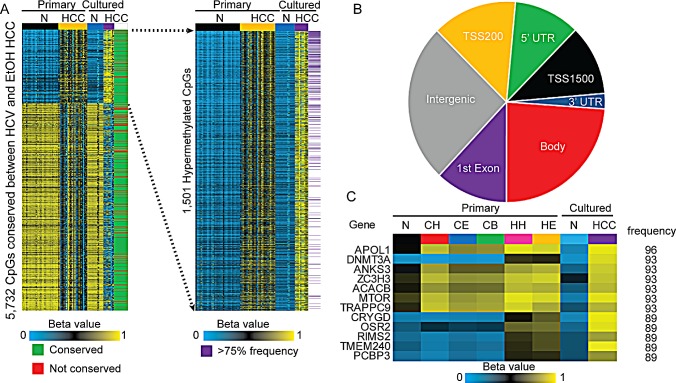
Conservation of hypermethylation and hypomethylation events between primary and cultured cells A. Heatmap depicting methylation levels of CpG sites conserved between primary HCC-HCV and HCC-EtOH, and whether they are conserved (green) or not conserved (red) in HCC cell lines. β-values for primary and cultured normal (N) and HCC are shown (FDR<0.05, Δβ>|0.25|). Blow-up of CpGs hypermethylated in HCC and the frequency with which they are hypermethylated in primary HCC samples (purple, >75% frequency). B. Classification of hypermethylation events conserved between primary and cultured HCC based on their location in the genome. C. Heatmap depicting the most frequently hypermethylated CpGs in primary HCC, the average β-values for primary normal liver (N), cirrhosis-HCV (CH), cirrhosis-EtOH (CE), cirrhosis-HBV (CB), HCC-HCV (HH), HCC-EtOH (HE) as well as cultured normal hepatocytes (N) and HCC cell lines. The frequency of hypermethylation in primary HCC is listed on the right.

Due to the large number of DNA methylation changes in HCC relative to normal liver, we sought to evaluate whether cell culture models mimic *in vivo* methylation changes, and therefore provide an experimental system in which the function of DNA methylation changes can be evaluated. We examined normal cultured hepatocytes (cultured for 3 days, *n*=15) and established liver cancer cell lines (*n*=10), resulting in Infinium 450k DNA methylation analysis of an additional 25 samples (Table 1, Supplemental Table 1,2). We limited the analysis to CpGs that were aberrantly methylated in both HCC-HCV and HCC-EtOH (primary tissues) relative to normal liver and compared them with the methylation changes that occur in the comparison between primary hepatocytes and established HCC cell lines. Of the 5,732 CpGs conserved between HCC-HCV and HCC-EtOH, over 85% of both hyper- and hypomethylation events were faithfully reflected in HCC cell lines relative to primary cultured hepatocytes (Δβ>|0.25|). Furthermore, CpGs that were hypermethylated in primary HCC and cultured tumor cells relative to their normal counterparts were highly conserved in primary samples (Figure 9A). Hypermethylation events were distributed throughout intragenic and intergenic features, with the gene body showing the most changes (Figure 9B). The twelve most frequently hypermethylated sites across all etiologies are predominantly located within the gene body (Figure 9C). One such example, *APOL1* displays copy number loss in 73.8% of ovarian cancers and 33.5% of tumors of the large intestine, but is not subject to genetic inactivation in liver cancer, based on the Catalogue of Somatic Mutations in Cancer (COSMIC, [46]). It is hypermethylated in 96% of the tumors analyzed in this study, however, suggesting that epigenetic deregulation of this gene, rather than mutation, could be a driver of liver disease. These CpGs are highly methylated in primary HCC regardless of clinical features, marking them as potentially useful disease markers that may be functionally involved in carcinogenesis or of value clinically. Indeed, these CpGs already show moderate hypermethylation in cirrhosis, which could be due to a small population of epigenetically poised pre-tumor cells with high levels of methylation, or increasing epigenetic instability within the entire population causing a graded increase in methylation in a large portion of diseased cells. Regardless, these findings suggest that epigenetic mis-regulation of these genes may contribute to liver tumorigenesis.

## DISCUSSION

**Figure 10:**
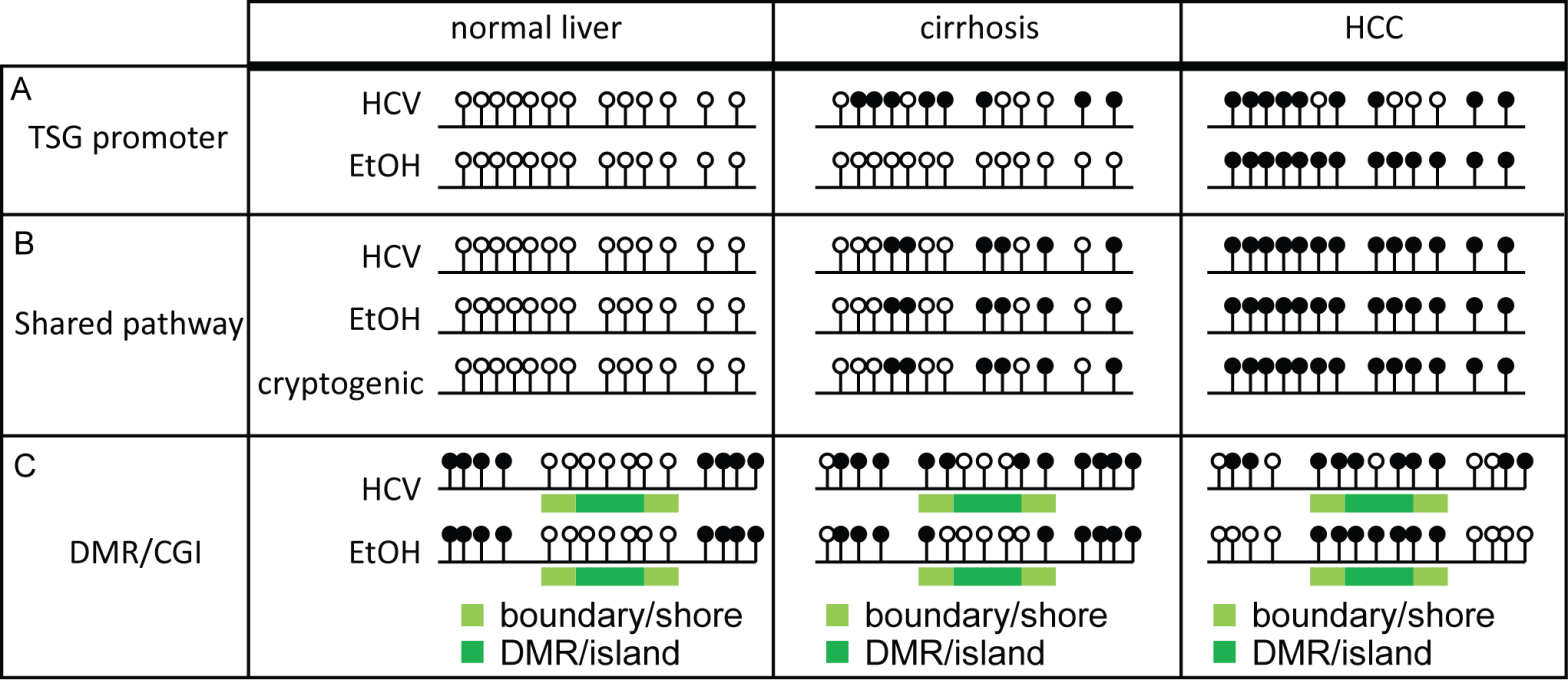
Schematic representation of DNA methylation changes during liver carcinogenesis A. Methylation of a tumor suppressor gene (TSG) in normal liver, cirrhosis, and HCC. Samples with HCV-infection display hypermethylation during cirrhosis, which is overtaken by changes induced by chronic alcoholism in HCC. B. Locus showing conserved methylation patterns between different etiologies, representing progressive biomarkers for hepatocarcinogenesis. C. Depiction of differentially methylated region (DMR) boundary erosion during liver disease and priming of CpG island (CGI) hypermethylation through CGI shore methylation during cirrhosis, especially under conditions of HCV-infection.

Widespread defects in DNA methylation, combined with multiple etiologic factors driving HCC, have been persistent confounding factors in elucidating the role of DNA methylation in HCC biology. In the largest study of its kind to-date, we sought to define etiology-specific and shared DNA methylation changes occurring across primary normal, cirrhotic, and HCC tissues and cell lines during liver disease progression. To this end, we demonstrated that: 1) HCV infection has a greater impact on DNA methylation during cirrhosis than other etiologies, 2) chronic alcoholism has a greater effect on the DNA methylation landscape than HCV infection in advanced liver disease (HCC), 3) HCC, regardless of etiology, manifests a substantially hypomethylated genome with large differentially methylated regions (DMRs; with ten times more hypomethylated DMRs relative to hypermethylated DMRs), 4) rare cirrhosis etiologies had relatively few epigenetic changes, while methylation changes in cryptogenic HCC substantially overlapped with HCC-HCV and HCC-EtOH, and 5) methylation changes observed in cirrhosis-HCV and conserved though HCC are associated with tumorigenic pathways (Figure 10A-C). These observations suggest a causal relationship between epigenetic lesions and liver disease progression, illustrating the need to perform further higher resolution genome-wide epigenomic studies on additional samples. Furthermore, genes robustly targeted for aberrant DNA methylation changes in cirrhosis and HCC represent potentially useful clinical markers of disease and/or epigenetic drivers of disease progression. Evaluation of the exact function of epigenetically deregulated genes will be the subject of future investigations.

A key objective of the current study was to gain insight into the overall epigenetic landscape of normal liver, cirrhosis, and HCC with different underlying etiologies. As seen in Figure 3D, the number of DNA methylation changes increases throughout liver carcinogenesis. Thus, recurrently hypermethylated loci across all samples independent of stage that were discovered in this study represent changes occurring relatively early during HCC progression making it likely that they are clinically relevant events. In this study, we observe hypermethylation of a subset of genes in more than 90% of primary HCC and HCC cell lines, independent of etiology (Figure 9). While the mutational frequency of these genes is low in HCC (1-2%), breast, ovarian, and lung cancers show frequent copy number loss at these loci, implying that epigenetic deregulation of these genes may substitute for genetic inactivation and influence the development and/or progression of liver disease. Indeed, among the notable hypermethylated genes unveiled in this study is *APOL1*, a member of a family of programmed cell death genes that initiates apoptosis, which has been demonstrated to be a biomarker for liver fibrosis [47, 48]. Furthermore, *APOL1* expression may be protective against renal cell carcinoma [49]. Overall, the consistently hypermethylated genes unveiled in this study not only represent potentially useful clinical markers of HCC, but also identify genes that, when epigenetically deregulated, may drive HCC initiation and progression. These genes therefore represent attractive candidates for future functional studies.

Currently sorafenib, a multi-kinase inhibitor, is the only FDA approved chemotherapeutic agent for liver cancer treatment. Sorafenib increases survival of patients with advanced HCC by only three months; no patients achieve complete remission [40]. Importantly, these studies were performed without regard to molecular phenotyping, which could mask beneficial effects on specific patient cohorts. Similarly, analysis of an unselected cohort of participants with advanced HCC in a phase II trial of the MET inhibitor tivantinib, showed no significant difference between placebo and drug-treated individuals; however, once patients were stratified into MET-high and MET-low groups, a positive difference in response was observed in the MET-high group [41]. This illustrates the need to delineate patient groups based on molecular signatures, potentially including DNA methylation landscapes, to improve treatment selection and response to therapy. Indeed, studies performed in an animal xenograft model with the DNA methyltransferase inhibitor zebularine demonstrated that stratification based on a “zebularine-sensitive” gene signature predicted the prognosis of patients with liver tumors [50]. Furthermore, a second generation DNA methyltransferase inhibitor, SGI-110, has recently been approved for a phase II clinical trial in patients resistant to sorafenib due to the ability of SGI-110 to demethylate and reactivate tumor suppressor genes in HCC cell lines [51]. The impact of these DNA methyltransferase inhibition studies are two-fold: first by emphasizing the potential benefit of patient stratification based on epigenetic biomarkers and molecular subtyping, and second by serving as proof-of-principle that DNA methyltransferase inhibitors may be beneficial in HCC treatment, which further underscores the important contribution of epigenetic deregulation to liver carcinogenesis. Our studies demonstrate that environmental factors such as chronic HCV infection or alcoholic liver disease affect the epigenome in disparate and overlapping ways and suggest that cirrhotic patients with HCV and chronic alcoholic patients with HCC may benefit from epigenetic therapy (Figure 10A,C).

The potential relevance of DNA methyltransferase inhibitors for cancer treatment is due in part to their ability to sensitize tumors through combination therapy [52]. DNA hypermethylation confers resistance to treatment of HCC cells by 5-fluorouracil through downregulation of the miR-193a-3p-SRSF2 axis [53]. Furthermore, loss of expression of mismatch repair (MMR) genes in late-stage HCC is associated with chemotherapy resistance as tumor cells become more tolerant of DNA damage in the absence of MMR gene expression [54, 55]. In addition, DNA methylation suppresses Jak/Stat pathway repressors, leading to unrestrained Jak/Stat pathway activation. Combination therapy with Jak/Stat inhibitors and the DNA methyltransferase inhibitor zebularine, however, leads to high levels of apoptosis [56]. The success of these therapies may in part be due to removal of aberrant hypermethylation at CpG islands within promoter regions of key cancer genes including *KLF4*, *RUNX3*, and *RSU1* identified as hypermethylated in our data [57-59]. Interestingly HCC-EtOH showed twice as many hypermethylation events relative to HCC-HCV, suggesting that alcoholic liver disease patients with HCC may respond more favorably to DNA methyltransferase inhibitor therapies than their HCV-infected counterparts assuming that hypermethylation is the key epigenetic lesion. It is interesting to note, however, that HCV infection in cirrhosis may predispose patients to HCC through an epigenetic mechanism, suggesting that DNA methylation inhibitors may be beneficial for perturbing the transition from cirrhosis to HCC (Figure 10A). Moreover, inhibition of DNA methylation may serve as adjuvant therapy to treat HCV-driven cirrhosis.

An important feature of our study was the ability to stratify samples based upon their etiology. We showed that with moderately high stringency, the majority of DNA methylation changes identified in cirrhotic tissues were hypermethylated specifically in the presence of HCV infection in regions flanking CpG islands (CpG shores and shelves, Figure 10C). Studies performed in mice with humanized livers infected with hepatitis B or hepatitis C virus correlated with our study in that DNA methylation changes were more frequent in livers infected with hepatitis C versus hepatitis B [60]. Moreover, many genes that displayed altered DNA methylation profiles in cirrhosis-HCV were conserved in HCC, suggesting these early epigenetic changes predispose HCV-infected cirrhosis patients to HCC. This is supported by the fact that HCV-infected individuals have a higher odds ratio for developing HCC than HBV-infected or alcoholic individuals [61]. Moreover, several clinical studies have demonstrated that sustained virologic response (SVR) to HCV therapy reduces but does not completely abrogate susceptibility to HCC, and indeed, cirrhotic patients that achieve SVR are still monitored for HCC development [62-64]. While there is a reduction in liver disease-related deaths by “curing” HCV infection, it is possible that perturbations in the epigenetic landscape during HCV-infection are maintained independent of HCV infection and predispose patients to disease progression (Figure 10A). It is therefore tempting to speculate that adjuvant therapy with DNA methyltransferase inhibitors may synergistically reduce the risk of HCC occurrence by removing aberrant DNA hypermethylation driven by previous HCV infection.

The field has recently begun to elucidate differentially methylated CpGs in HCC as well as the influence of etiologic factors such as aflatoxin, using targeted and array-based approaches [29, 65, 66]. Consistent with previously published data obtained with the Infinium 27k and 450k HumanMethylation BeadChip, a significant proportion of hypomethylation or hypermethylation events identified are reflected in our data set irrespective of etiology (Figure 10B and data not shown, [67-69]). We and others have found that many genes are coordinately epigenetically deregulated, either through hypermethylation or hypomethylation, during the progression of disease from cirrhosis to HCC [29, 31]. For example, the Wnt pathway negative regulator *SFRP3* is hypermethylated in a stepwise fashion from normal liver to hepatitis through cirrhosis and finally HCC [70]. In addition, candidate gene approaches identifying frequently hypermethylated genes in HCC, such as *CDKN2A*, *CDKN2B*, and *GSTP1* are faithfully reproduced in our study (data not shown, [71-73]). Recently, it has been shown that gene body methylation correlates with activation and may be a therapeutic target in cancer [74]. Many CpG sites with conserved methylation changes between cirrhosis-HCV and HCC showed aberrant methylation not only within promoter regions, but also in gene bodies. Moreover, it has been postulated that exons that are methylated are more likely to be included in mRNA transcripts [43, 75]. In addition to control of exon inclusion, gene body DNA methylation may play a role in controlling alternative promoter usage, as many genes have multiple transcription start sites [76]. Our study has revealed that large domains of DNA are hypermethylated or hypomethylated in a stepwise manner through liver disease progression (Figure 10C). For example, the 60,000 base pair HOXA locus is not only frequently hypermethylated in HCC, but appears to be methylated in a stepwise process originating during cirrhosis (Supplemental Figure 5,[77]). Thus, while the functional significance of gene body methylation requires further research, regions like the HOXA and SUSD4 locus identified in this study can provide a snapshot of disease progression and perhaps assist in cancer surveillance during the progression to HCC from cirrhosis or apparently normal liver.

With the exception of HBV integration into the hepatocyte genome, there are relatively few consistent genetic aberrations in HCC. The most prevalent genetic changes identified from multiple studies include mutations in the TERT promoter (59.0% of HCCs tested), *β-catenin* (oncogene, 11.1-15.9%), and *TP53* (tumor suppressor, 35.2-51.8%)[78-80]. Interestingly, epigenetic modifying enzymes such as *ARID2* and *MLL* are recurrently mutated in HCC [80, 81]. Pan-cancer analysis of somatic mutation data has revealed five mutational “subtypes” for liver cancer, suggesting that liver cancer is a heterogeneous disease, perhaps due to the influence of individual etiologies [82]. Indeed, one of the twenty-six mutational signatures identified from the pan-cancer analysis was attributed specifically to aflatoxin exposure in liver cancer [82]. Furthermore, the *IRF2* gene has recently been shown to be mutated exclusively in HCC-HBV [83]. Overall, it has been postulated that cellular selection and downstream growth advantages are attributable to aberrant epigenetic changes, and this is most likely true in the absence of frequent somatic mutations [84-86]. Due to the lower frequency of common mutations in combination with varying epigenetic dysregulation based on etiology, it appears that HCC arises through a complex interaction of genetic, epigenetic, and environmental factors. While data presented in this study may provide epigenetic signatures that can one day be used to better stratify patient response to therapy as well as provide epigenetic biomarkers for liver disease progression, it stresses the necessity for future integrated genetic and epigenetic approaches to fully understand the process of liver carcinogenesis.

## METHODS

### Human tissues, primary cultures, and HCC cell lines

Primary livers from four patients were perfused, hepatocytes were isolated, plated, and cultured as previously described to yield short term cultures of normal primary hepatocytes (n>=2 cultures per patient) [87]. Tumor cell lines were acquired from ATCC or grown from primary human tumors removed by surgery at the University of Florida using standard cell culture procedures (Supplemental Table 1,2). Cirrhotic and HCC samples were obtained by surgical resection at the University of Florida Shands Hospital. Normal livers were obtained from patients undergoing surgery for colorectal carcinoma metastases to the liver or benign liver lesions such as benign cysts or hemangiomas. Tissues were snap frozen and stored at −135°C. The tissue collection protocol is approved by the Institutional Review Board and patient consent.

### DNA Methylation assays

Genomic DNA was isolated and checked for quality by standard protocols prior to bisulfite treatment using the EZ DNA Methylation Kit (Zymo, Irvine, CA) and hybridized to the Infinium 450k HumanMethylation BeadChip (Illumina, San Diego, CA) according to the manufacturer's specifications. Bisulfite sequencing was performed as previously described [88]. Briefly, PCR fragments amplified from bisulfite treated DNA were ligated into a TA vector (Invitrogen, Carlsbad, CA). DNA isolated from plasmids was sequenced at the ICBR Core Facility at the University of Florida. Resultant sequencing data was analyzed using QUMA (http://quma.cdb.riken.jp/, [89]). Primer sequences are listed in Supplemental Table 3.

### Data processing and statistics

Quality control of Infinium 450k Human Methylation BeadChips was performed via the Genome Studio Methylation Module (Illumina). Subset-quantile Within Array Normalization (SWAN) was performed on the Infinium 450k Human Methylation BeadChip IDAT files via the R Bioconductor package “minfi” [90]. The resultant beta values (β) were annotated based on the manufacturer's recommendation and supplemented with advanced annotation [91]. Infinium 450k data is available through NCBI GEO (accession: GSE60753). Student's *t* tests (p-values) were applied to normalized beta values and Benjamini-Hochberg adjustments (FDR-values) were used to account for multiple testing. All relevant 450k analyses had a cutoff of FDR<0.05 with the indicated methylation change (Δβ). Heat maps from processed data were also created though R (“heatmap.2”) or custom macros in Microsoft Excel. Genomic views of loci were prepared using the UCSC genome browser as bedGraphs [92]. Bisulfite sequencing data was compared using Fisher's exact tests, with a p-value <0.05 considered significant. Relationships between gene features were determined by correlation coefficients and regression analysis, with p<0.05 considered significant. Tag density plots include 5,000 base pairs flanking the gene, with the gene body data normalized to a percentage of gene length. Gene expression data from seven normal livers was acquired from NCBI GEO (accession: GSE28619, [37]). Bioconductor was used to annotate probes using hgu133plus2.db and gene expression was calculated by averaging the signal intensities of probes for each gene using an in-house script.

## SUPPLEMENTARY MATERIAL, TABLE AND FIGURES


