# Supplementary material for: Epigenetic signatures of alcohol abuse and hepatitis infection during human hepatocarcinogenesis
Source: Oncotarget. 2014 Sep 8;5(19):9425–43. doi: 10.18632/oncotarget.2444 (PMC4253444; doi:10.18632/oncotarget.2444)
Supplement: Supplementary file 1 [file oncotarget-05-9425-s001.pdf]

Epigenetic signatures of alcohol abuse and hepatitis infection during human hepatocarcinogenesis

Supplementary Material

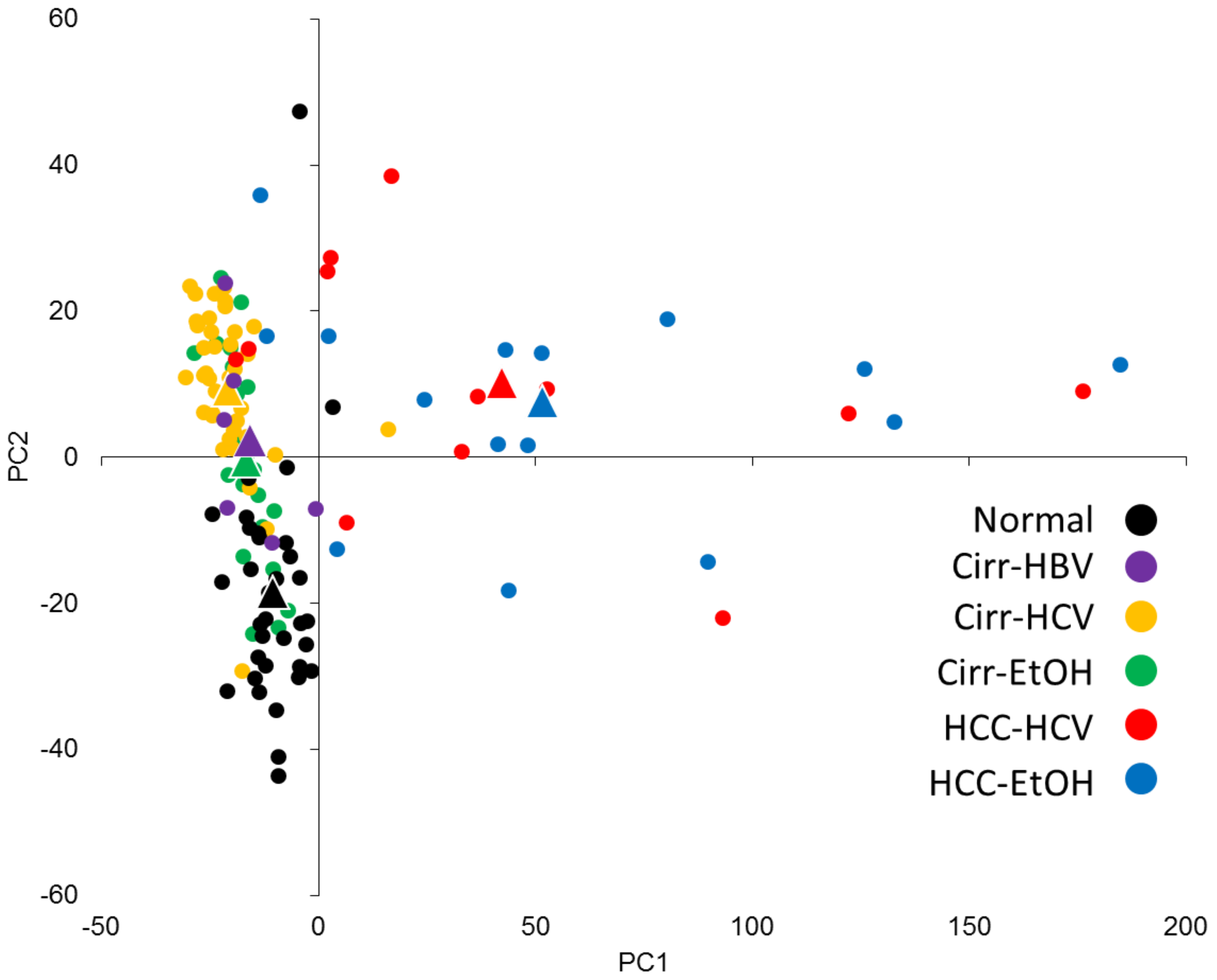

Supplemental Figure 1: Principal component analysis. Individual samples are denoted by circles and group averages represented by triangles of the same color.

A

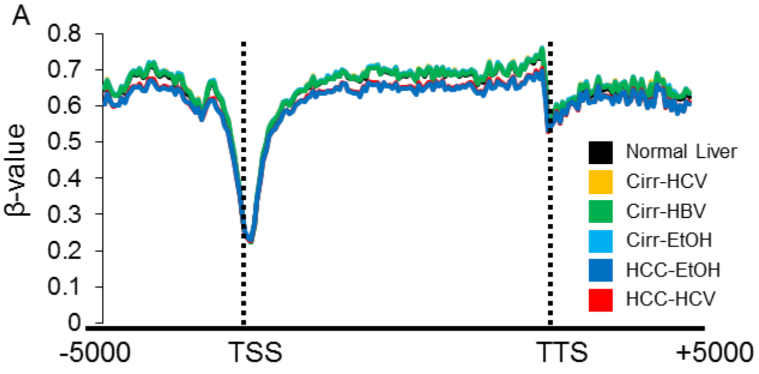

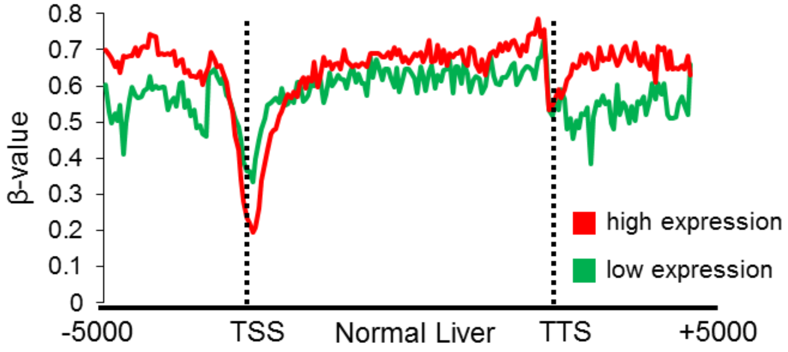

B

HCV	TSS1500	TSS200	5'UTR	1st Exon	Body	3'UTR
TSS1500	1	0.650561	0.521984	0.609813	0.422344	0.264464
TSS200		1	0.648679	0.791122	0.3434	0.122484
5'UTR			1	0.588881	0.380223	0.22293
1st Exon				1	0.308474	0.112521
Body					1	0.546036
3'UTR						1

EtOH	TSS1500	TSS200	5'UTR	1st Exon	Body	3'UTR
TSS1500	1	0.671742	0.534208	0.631657	0.436082	0.270227
TSS200		1	0.671082	0.81259	0.349584	0.112819
5'UTR			1	0.60837	0.378861	0.216843
1st Exon				1	0.314428	0.107761
Body					1	0.564449
3'UTR						1

Expression stratification

HCV_up	TSS1500	TSS200	5'UTR	1st Exon	Body	3'UTR
TSS1500	1	0.449051	0.374844	0.377411	0.222029	0.094558
TSS200		1	0.581797	0.636493	0.177888	0.043771
5'UTR			1	0.449822	0.228975	0.099427
1st Exon				1	0.153448	0.039663
Body					1	0.292927
3'UTR						1

EtOH_up	TSS1500	TSS200	5'UTR	1st Exon	Body	3'UTR
TSS1500	1	0.478506	0.420288	0.420993	0.224965	0.079269
TSS200		1	0.631778	0.697182	0.204521	0.030021
5'UTR			1	0.486204	0.232679	0.063332
1st Exon				1	0.16856	0.019162
Body					1	0.28898
3'UTR						1

HCV_down	TSS1500	TSS200	5'UTR	1st Exon	Body	3'UTR
TSS1500	1	0.702824	0.611103	0.661427	0.432147	0.275745
TSS200		1	0.7	0.807384	0.377936	0.161566
5'UTR			1	0.619361	0.448531	0.26806
1st Exon				1	0.369981	0.168575
Body					1	0.568723
3'UTR						1

EtOH_down	TSS1500	TSS200	5'UTR	1st Exon	Body	3'UTR
TSS1500	1	0.725038	0.623699	0.674107	0.458504	0.282577
TSS200		1	0.712301	0.821022	0.404594	0.180894
5'UTR			1	0.627437	0.460677	0.273889
1st Exon				1	0.386904	0.197343
Body					1	0.593981
3'UTR						1

Supplemental Figure 2: Correlation analysis across intragenic elements. A. DNA methylation β-values for normal liver, cirrhosis etiologies, and HCC etiologies relative to position within genes from 5,000 base pairs upstream of the gene (-5,000), through the transcription start site (TSS), the transcription termination site (TTS) and 5,000 base pairs downstream of the gene (+5,000)[top]. Distribution of β-values across genes in the normal liver for highly expressed genes (red) and lowly expressed genes (green) [bottom]. C. Correlation coefficients between DNA methylation in intragenic features for genes in HCC-HCV (left) or HCC-EtOH (right). Correlations coefficients for all genes based on genetic features in HCC-HCV and HCC-EtOH (top), the 25% highest expressed genes ("up", middle), and the 25% lowest expressed genes ("down", bottom).

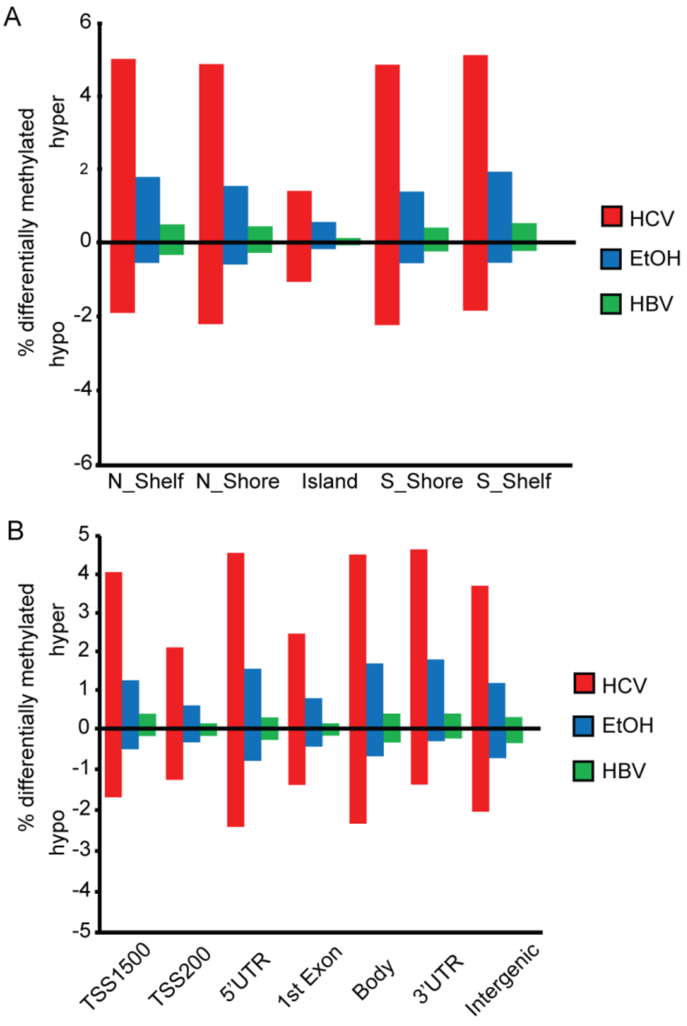

Supplemental Figure 3: Distribution of methylation changes across the genome in liver cirrhosis. A. Bar chart representing the percentage of hypermethylation (positive values) and hypomethylation (negative values) changes across CpG island features, including north shelves (N_Shelf), north shores (N_Shore), islands, south shores (S_Shore), and south shelves (S_Shelf) in cirrhosis-EtOH (blue), cirrhosis-HBV (green), and cirrhosis-HCV (red) relative to the total number of each feature on the Infinium 450k array. ($p<0.05$, $\Delta\beta>|0.2|$). B. Bar chart for intergenic features.

A

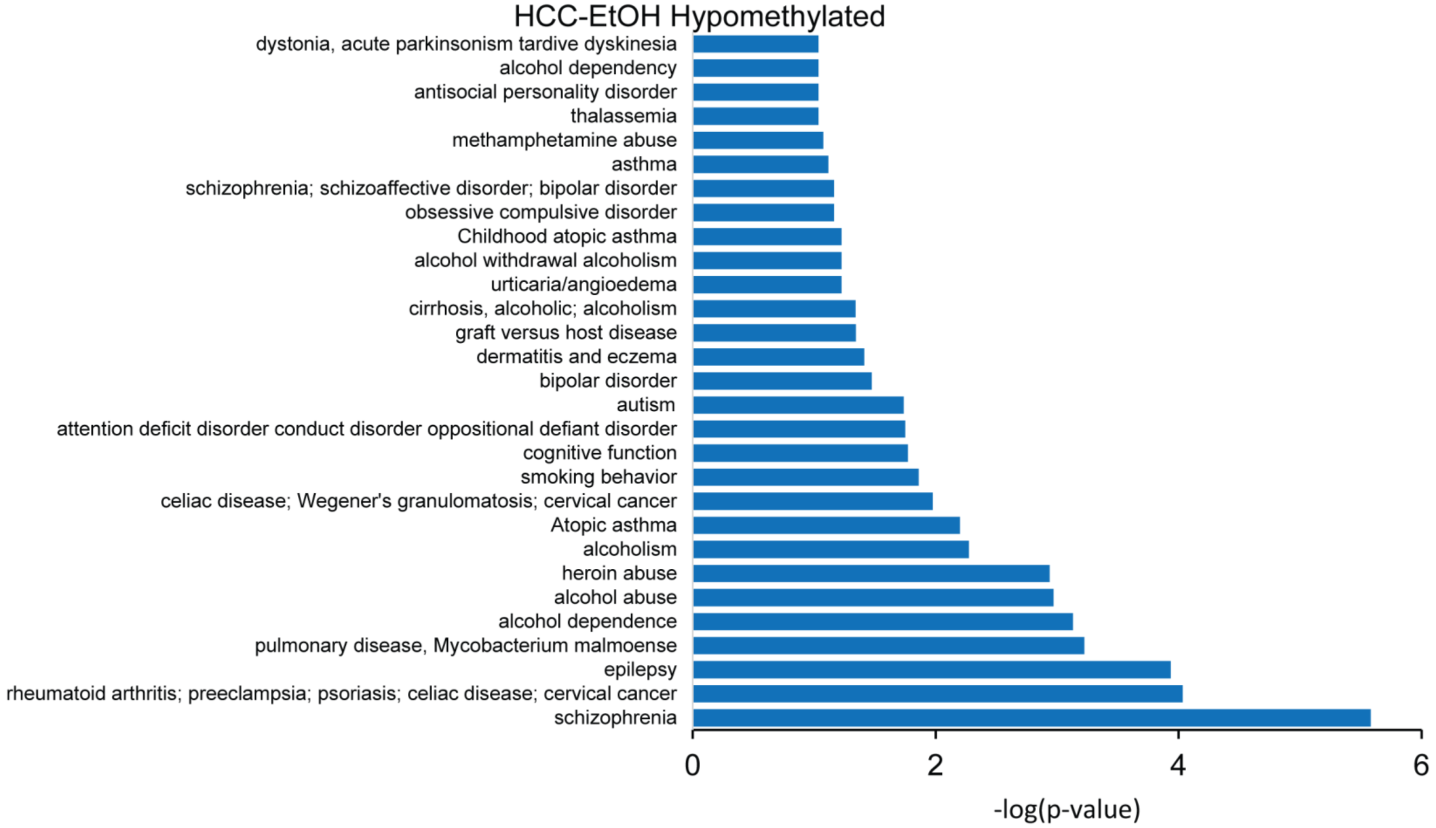

B

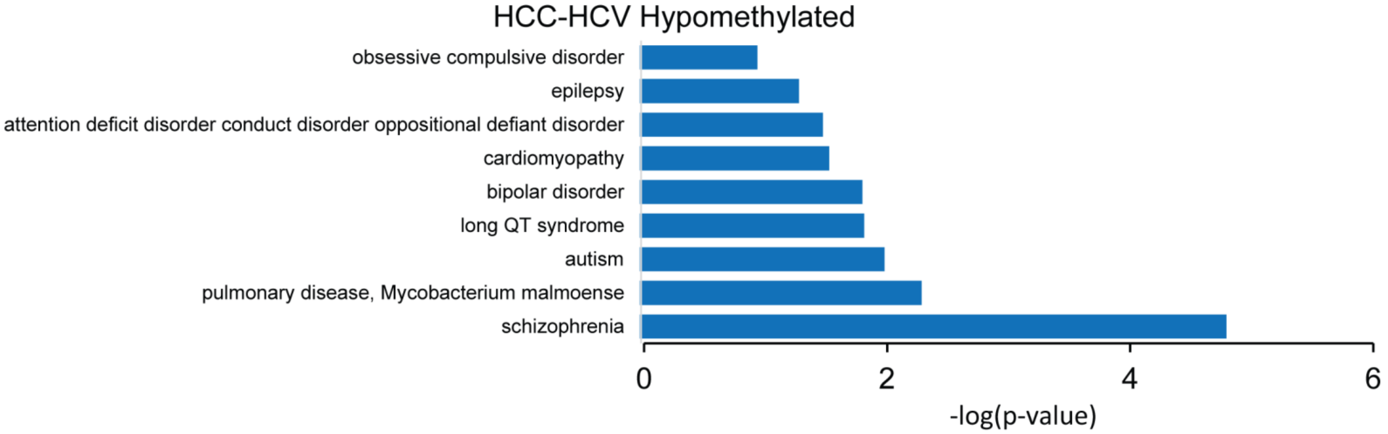

Supplemental Figure 4: Functional pathways associated with DNA methylation changes in chronic HCV-infection and alcohol abuse-related HCC. Bar charts depicting significance of DAVID ontology analysis from CpGs associated with hypomethylated genes in HCC-EtOH (A, Δβ<-0.25) or hypomethylated genes in HCC-HCV (B, Δβ<-0.25).

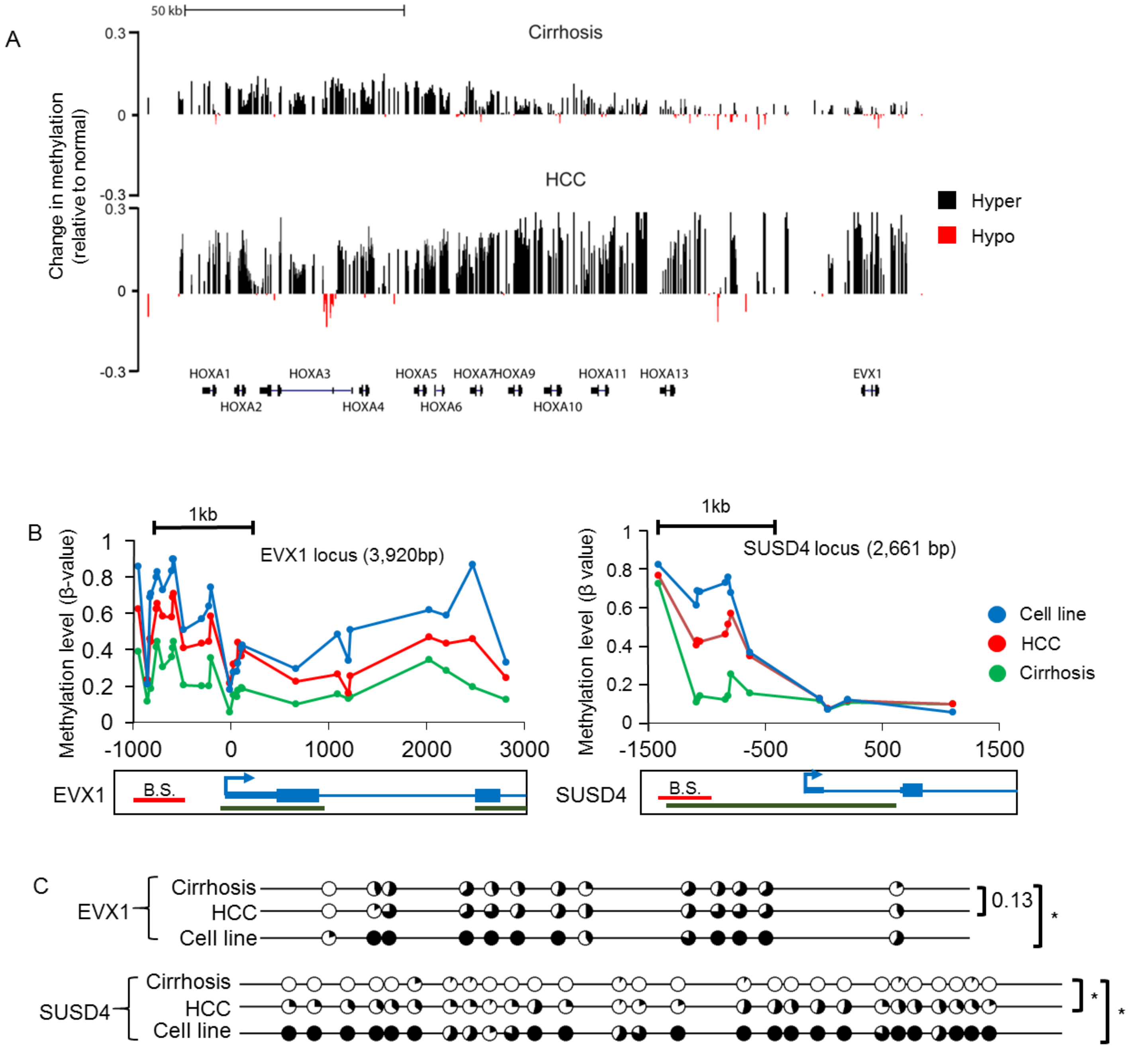

Supplemental Figure 5: Stepwise regional hypermethylation during liver disease progression. A. UCSC genome browser shot depicting the 60kb HOXA locus. Black (hypermethylated) and red (hypomethylated) bars represent probes on the 450k array that are significantly changed in cirrhosis (top) and HCC (bottom) relative to normal liver. B. Schematic representation of the EVX1 and SUSD4 methylation levels in HCC cell lines (blue), primary HCC samples (red) and primary cirrhotic liver samples (green) based on Infinium 450k data. The position for primers for bisulfite sequencing is depicted by a red bar (B.S.) with CpG islands as a dark green bar. C. Bisulfite sequencing of up to 12 clones in cirrhosis, HCC, and the HCC cell line HCO2 for SUSD4 (top) and EVX1 (bottom). Each wedge of the pie represents one allele's methylation status, methylation and lack of methylation in black and white, respectively. P-values for comparisons are shown on the right (Fisher's exact test, *p<0.05).

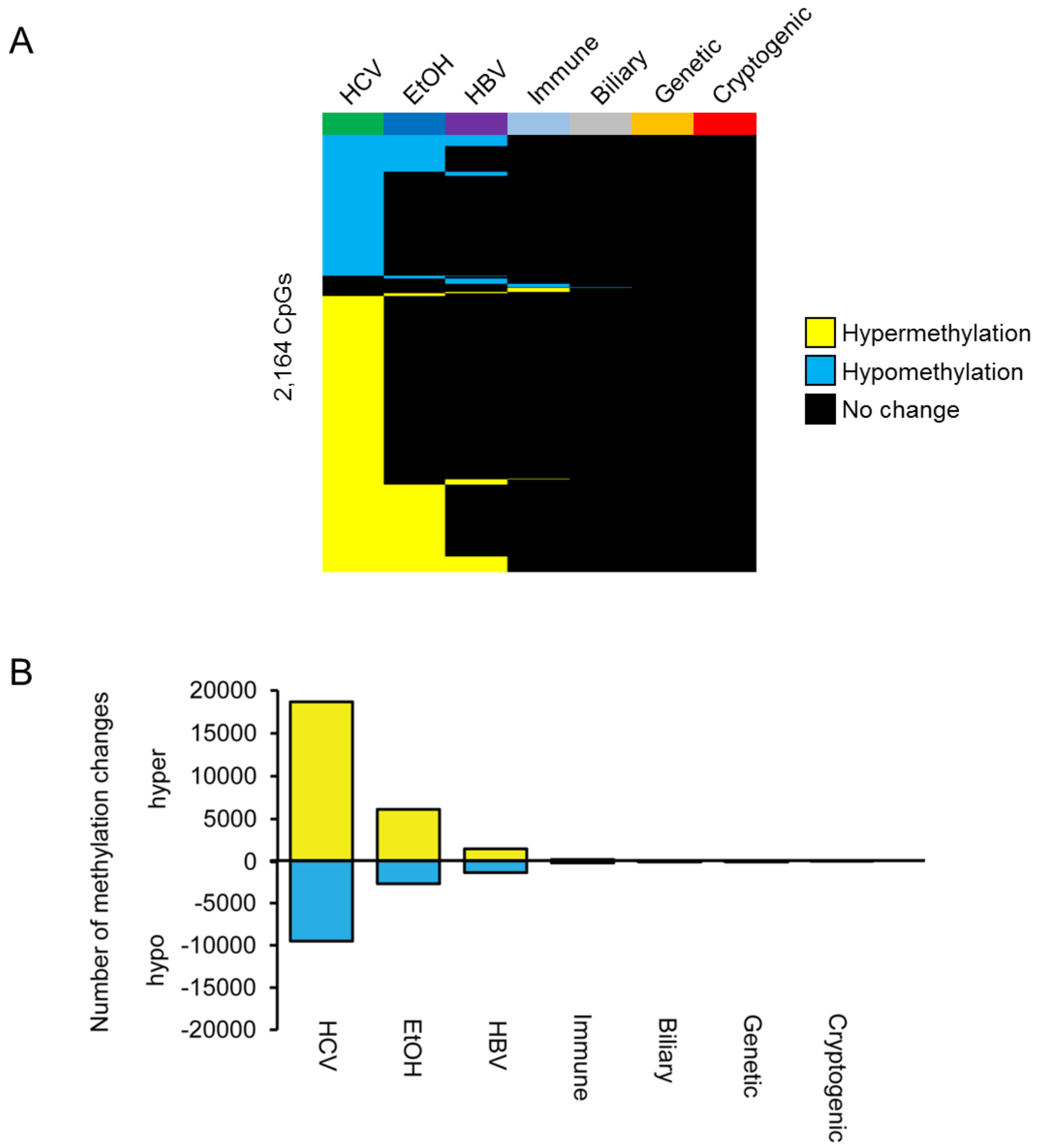

Supplemental Figure 6: Analysis of DNA methylation in minor cirrhosis etiologies. A. Heatmap of statistically significant changes in cirrhotic patients with hepatitis C infection (HCV), chronic alcoholism (EtOH), hepatitis B infection, (HBV), genetically driven causes (Genetic), cryptogenic cirrhosis (Crypto), immune deficiencies (Immune), and impaired biliary function (Biliary) relative to normal liver. Hypermethylation is shown in yellow, hypomethylation in blue, no change in black ($p<0.05$, $\Delta\beta>|0.25|$). B. Bar graph depicting the number of hypermethylation (yellow, positive) and hypomethylation (blue, negative) events in each of the cirrhosis etiologies relative to normal liver.

Supplementary Table 1: Summary of human liver samples analyzed in this study. The total number of samples, their mean age with the standard deviation (SD), and gender for normal, cirrhotic, and liver tumor samples. Cultured cells used are also shown. Characterization based on etiology is shown for hepatitis C infection (HCV), hepatitis B infection (HBV), chronic alcoholism (EtOH) and rare/cryptogenic (Other) samples.

**Group**	**Number**	**Age (S.D)**	**Male**	**Female**
Normal	34	55.5 ± 17.8	17	17
Cirrhosis	77	55.1 ± 9.6	54	23
HCV	39	53.6 ± 7.9	26	13
HBV	6	58.6 ± 4.3	5	1
EtOH	21	58.0 ± 9.7	17	4
Other	11	53.1 ± 14.8	6	5
Cancer	45	61.0 ± 11.4	31	14
HCV	12	56.2 ± 7.1	9	3
HBV	2	60.5 ± 7.8	1	1
EtOH	15	66.3 ± 8.2	12	3
Other	16	59.1 ± 14.5	8	8
Cultured Cells	25			
Normal Hepatocytes	15			
HCC cell lines	10			

Supplemental Table 2: Sample classification for less frequent cirrhosis and cancer samples and established cell cultures. Characterization based upon the disease stage, etiology, and the category for downstream analysis.

Group	Description	Category
Cirrhosis	AAT deficiency	Genetic
Cirrhosis	AAT deficiency	Genetic
Cirrhosis	Autoimmune hepatitis	Immune
Cirrhosis	Biliary obstruction	Biliary
Cirrhosis	Cryptogenic	Cryptogenic
Cirrhosis	Cryptogenic	Cryptogenic
Cirrhosis	Cryptogenic	Cryptogenic
Cirrhosis	Hemochromatosis	Genetic
Cirrhosis	Hemochromatosis	Genetic
Cirrhosis	Primary biliary	Biliary
Cirrhosis	Sarcoidosis	Immune
HCC	Cholangio carcinoma	Biliary
HCC	Cholangio carcinoma	Biliary
HCC	Colon cancer metastasis	Metastatic
HCC	Colon cancer metastasis	Metastatic
HCC	Lymphoma metastasis	Metastatic
HCC	Lymphoma metastasis	Metastatic
HCC	Melanoma metastasis	Metastatic
HCC	Neuroendocrine metastasis	Metastatic
HCC	Sarcoma metastasis	Metastatic
HCC	Squamous carcinoma metastasis	Metastatic
HCC	Cryptogenic	Cryptogenic
HCC	Cryptogenic	Cryptogenic
HCC	Cryptogenic	Cryptogenic
HCC	Cryptogenic	Cryptogenic
HCC	Cryptogenic	Cryptogenic
HCC	Cryptogenic	Cryptogenic
HCC	Cell Line	Huh7
HCC	Cell Line	FLNEO
HCC	Cell Line	H801
HCC	Cell Line	HCO2
HCC	Cell Line	Hep3B
HCC	Cell Line	Huh75
HCC	Cell Line	Huh75-JHF1
HCC	Cell Line	LH86
HCC	Cell Line	SNU423
HCC	Cell Line	SNU449

Supplemental Table 3: Primers used in this study. Forward (F) and Reverse (R) oligonucleotides used for amplification of bisulfite DNA for bisulfite sequencing of the EVX1 and SUSD4 loci.

ID	Sequence
EVX1-F	TTTGGGAGAGGATTTTATGAGTTAA
EVX1-R	TTTTCTTCCCTAAAAAACAAAAAATAA
SUSD4-F	TTTTTTTTATATTTTTGGTATTTTTTTT
SUSD4-R	TCATTAACTAACACCTCTTAATCACTATAA
